# Characterization of *Vibrio parahaemolyticus* clinical strains from Maryland (2012–2013) and comparisons to a locally and globally diverse *V. parahaemolyticus* strains by whole-genome sequence analysis

**DOI:** 10.3389/fmicb.2015.00125

**Published:** 2015-02-19

**Authors:** Julie Haendiges, Ruth Timme, Marc W. Allard, Robert A. Myers, Eric W. Brown, Narjol Gonzalez-Escalona

**Affiliations:** ^1^Department of Health and Mental HygieneBaltimore, MD, USA; ^2^Center for Food and Applied Nutrition, Food and Drug AdministrationCollege Park, MD, USA

**Keywords:** NGS, WGS, *Vibrio parahaemolyticus*, clinical, phylogenetic analysis, phylogeny, SNPs

## Abstract

*Vibrio parahaemolyticus* is the leading cause of foodborne illnesses in the US associated with the consumption of raw shellfish. Previous population studies of *V. parahaemolyticus* have used Multi-Locus Sequence Typing (MLST) or Pulsed Field Gel Electrophoresis (PFGE). Whole genome sequencing (WGS) provides a much higher level of resolution, but has been used to characterize only a few United States (US) clinical isolates. Here we report the WGS characterization of 34 genomes of *V. parahaemolyticus* strains that were isolated from clinical cases in the state of Maryland (MD) during 2 years (2012–2013). These 2 years saw an increase of *V. parahaemolyticus* cases compared to previous years. Among these MD isolates, 28% were negative for *tdh* and *trh*, 8% were *tdh* positive only, 11% were *trh* positive only, and 53% contained both genes. We compared this set of *V. parahaemolyticus* genomes to those of a collection of 17 archival strains from the US (10 previously sequenced strains and 7 from NCBI, collected between 1988 and 2004) and 15 international strains, isolated from geographically-diverse environmental and clinical sources (collected between 1980 and 2010). A WGS phylogenetic analysis of these strains revealed the regional outbreak strains from MD are highly diverse and yet genetically distinct from the international strains. Some MD strains caused outbreaks 2 years in a row, indicating a local source of contamination (e.g., ST631). Advances in WGS will enable this type of analysis to become routine, providing an excellent tool for improved surveillance. Databases built with phylogenetic data will help pinpoint sources of contamination in future outbreaks and contribute to faster outbreak control.

## Introduction

*Vibrio parahaemolyticus* is a natural inhabitant of temperate and tropical coastal waters and is the leading cause of seafood-borne gastroenteritis in the United States (US) (Scallan et al., 2011). Cases of illness are usually associated with eating raw or undercooked seafood. Strains of *V. parahaemolyticus* carrying genes for thermostable direct hemolysin (*tdh*) and/or thermostable direct hemolysin-related hemolysin (*trh*) are considered pathogenic (Turner et al., 2013) and typically represent <1% of environmental *V. parahaemolyticus* strains (DePaola et al., 2000). However, this frequency may depend on the location, sample sources, and detection methods (Kaysner et al., 1990; Alam et al., 2002; Cook et al., 2002; Hervio-Heath et al., 2002; Martinez-Urtaza et al., 2008). During the last two decades *V. parahaemolyticus* infections and outbreaks have increased throughout the world. Most of these new cases belong to a pandemic clonal complex, known as CC3, first identified in in February of 1996 in India (Martinez-Urtaza et al., 2005; Nair et al., 2007; Gonzalez-Escalona et al., 2008; Haendiges et al., 2014).

The emergence of CC3 caused public health concerns about the potential worldwide spread of virulent *V. parahaemolyticus*, which previously had been restricted to particular regions. Other clonal complexes of *V. parahaemolyticus*, specifically CC36 and CC34, have been observed among coastal US strains (Gonzalez-Escalona et al., 2008), and strains of Sequence Type (ST) 36, the ancestral type of CC36, have also been detected on the western coast of Canada (Banerjee et al., 2014). Although infections in the US are typically caused by strains from CC36, which is endemic to the West Coast (Abbott et al., 1989; Gonzalez-Escalona et al., 2008), a Maryland outbreak in August 2012 (Haendiges et al., 2014) was caused by strains belonging to CC3.

Foodborne illnesses due to *V. parahaemolyticus* are uncommonly reported in the US and in the state of Maryland. Forty-six cases of *V. parahaemolyticus* gastroenteritis associated illnesses were reported between 2012 and 2013. From those cases, 34 strains were isolated. During the summer of 2012 a multistate outbreak associated with harvested shellfish was reported on the East Coast of the US (Newton et al., 2014), caused by *V. parahaemolyticus* strains belonging to CC36 (Martinez-Urtaza et al., 2013), subsequently shown to be ST36. That outbreak affected 28 persons in nine states (Newton et al., 2014), and the isolated strains were of the O4:K12 or O4:K (unknown) serotypes. Usually strains from this serotype/ST isolated in the US are from the West Coast (Gonzalez-Escalona et al., 2008; Newton et al., 2014). Another outbreak, involving 104 cases, occurred in the summer of 2013 affecting people in 13 US states and were caused by the same ST36 (Newton et al., 2014). An outbreak in Spain, during the summer of 2012, was attributed to strains that were ST36 as well (Martinez-Urtaza et al., 2013), associated with cooked seafood that had been cooled with untreated local seawater (Martinez-Urtaza et al., 2013); contrarily to the infections in the US which are often linked to the consumption of contaminated oysters (Newton et al., 2014).

The numbers of *V. parahaemolyticus* infections appears to have increased approximately 4 times in US in the last decade (Mead et al., 1999; Scallan et al., 2011). Scallan et al. (2011) reported that the domestically acquired foodborne average for *V. parahaemolyticus* infections annually were around 35,000 in the period 2000–2008, while Mead et al. (1999) reported around 8000 infections annually in the period 1992–1997. The increasing number of infections could have detrimental impacts on public health and economic growth, particularly in regions where seafood harvest and consumption are important. However, active surveillance depends upon having effective ways to identify and monitor the nature or identity of the *V. parahaemolyticus* strains causing outbreaks.

Various typing methods have been used to distinguish bacterial isolates for epidemiological investigations (Foxman et al., 2005). Pulsed Field Gel Electrophoresis (PFGE) has been a favored method for genotyping *V. parahaemolyticus* isolates (Marshall et al., 1999) and it is considered the “gold standard” for outbreak investigations (Parsons et al., 2007; Wagley et al., 2008; Dauros et al., 2011; Banerjee et al., 2014; Haendiges et al., 2014; Ma et al., 2014; Pazhani et al., 2014). While useful for short-term epidemiology, PFGE does not provide details of the genetic relationships among isolates (e.g., evolutionary relationships) (Foxman et al., 2005). Another common method for characterizing *V. parahaemolyticus* isolates is Multilocus Sequence Typing (MLST). This method is based on direct sequence analysis of housekeeping genes, making MLST better for long term evolutionary studies (Gonzalez-Escalona et al., 2008; Harth et al., 2009; Yan et al., 2011; Gavilan et al., 2013; Martinez-Urtaza et al., 2013; Turner et al., 2013). A public database was established to archive *V. parahaemolyticus* sequences (http://pubmlst.org/vparahaemolyticus) (Gonzalez-Escalona et al., 2008).

In the last 5 years, scientists have begun using genomic techniques to analyze historical collections of pathogens and outbreak isolates, providing new insights for outbreak investigations. Whole genome sequencing (WGS) and WGS-SNP data analyses allow us to better understand population dynamics and mechanisms contributing to increased virulence among foodborne bacterial pathogens, including outbreaks of *Salmonella* Montevideo in 2010 (Bakker et al., 2011; Allard et al., 2012), *Vibrio cholerae* in Haiti in 2010 (Chin et al., 2011), *E. coli* O104:H4 (in Germany in 2011, Rasko et al., 2011) and *Salmonella* Enteritidis in the US in 2010 (Allard et al., 2013). In the current project, we utilize a similar approach to explore the diversity and relationships among *V. parahaemolyticus* isolates causing outbreaks in Maryland.

Specifically, in order to better understand potential changes in *V. parahaemolyticus* populations in the state of Maryland and to investigate whether the spikes in cases during the summers of 2012 and 2013 were related to this recently introduced East Coast clone, ST36, we used WGS to compare genomes from those outbreaks with those of the other *V. parahaemolyticus* strains causing illnesses in recent years (2012–2013). We began by sequencing the 34 strains from MD, then 10 additional historical *V. parahaemolyticus* strains from different sources (clinical and environmental) from the East and West Coasts of US were also sequenced and used for phylogenetic comparative analysis. Additional available genomes from global *V. parahaemolyticus* strains were also used in the phylogenetic comparative analysis.

## Materials and methods

### Bacterial strains and media

The *V. parahaemolyticus* isolates sequenced for this project are listed, along with their assigned CFSAN numbers, in Tables 1, 2. Table 1 lists the 34 clinical isolates from Maryland, and Table 2 lists the national and international *V. parahaemolyticus* isolates used for comparison. All isolates were retrieved from storage (−80°C freezer), transferred to Luria-Bertani (LB) medium with 3% NaCl and incubated at 250 rpm at 37°C.

**Table 1:** **Clinical *V. parahaemolyticus* isolates sequenced in this study and source information**.

**Isolate name**	**Accession no(s) WGS**	**CFSAN number**	**STs**	**County**	**State**	**Collection date**	**Source**	***tdh, trh* presence^a^**	**References**
VP1	JNSM01000000	CFSAN007429	631	Anne Arundel	MD	6/15/2012	stool	2,1	Haendiges et al., 2014
VP8	JNSN01000000	CFSAN007430	631	Cecil	MD	7/12/2012	stool	2,1	Haendiges et al., 2014
VP9	JNSO01000000	CFSAN007431	631	Queen Anne's	MD	7/17/2012	stool	2,1	Haendiges et al., 2014
VP31	JNSP01000000	CFSAN007432	631	Harford	MD	6/16/2013	stool	2,1	Haendiges et al., 2014
VP35	JNSQ01000000	CFSAN007433	631	Baltimore City	MD	7/11/2013	stool	2,1	Haendiges et al., 2014
VP41	JNSR01000000	CFSAN007434	631	Unknown	MD	7/17/2013	stool	2,1	Haendiges et al., 2014
VP44	JNSS01000000	CFSAN007435	631	Unknown	MD	8/3/2013	stool	2,1	Haendiges et al., 2014
VP45	JNST01000000	CFSAN007436	631	Anne Arundel	MD	8/9/2013	stool	2,1	Haendiges et al., 2014
VP2	JNSU01000000	CFSAN007437	651	Montgomery	MD	5/31/2012	stool	0,0	Haendiges et al., 2014
VP4	JNSW01000000	CFSAN007439	653	Out of State-DE	MD	6/17/2012	stool	0,2	Haendiges et al., 2014
VP34	JNSX01000000	CFSAN007440	653	Prince George's	MD	7/11/2013	stool	0,2	Haendiges et al., 2014
VP7	JNSZ01000000	CFSAN007442	113	Charles	MD	7/10/2012	stool	2,1	Haendiges et al., 2014
VP10	JNTC01000000	CFSAN007445	43	Montgomery	MD	6/12/2012	stool	2,1	Haendiges et al., 2014
VP16	JNTG01000000	CFSAN007449	3	Anne Arundel	MD	8/21/2012	stool	1 and 2, 0	Haendiges et al., 2014
VP17	JNTH01000000	CFSAN007450	3	Anne Arundel	MD	8/22/2012	stool	1 and 2, 0	Haendiges et al., 2014
VP18	JNTI01000000	CFSAN007451	3	Anne Arundel	MD	8/24/2012	stool	1 and 2, 0	Haendiges et al., 2014
VP39	JNTL00000000	CFSAN007455	896	Wicomico	MD	7/19/2013	stool	0,0	Haendiges et al., 2014
VP12	JNTM00000000	CFSAN006129	36	Montgomery	MD	8/3/2012	stool	2,1	Haendiges et al., 2014
VP32	JNTN00000000	CFSAN006131	36	Anne Arundel	MD	6/30/2013	stool	2,1	Haendiges et al., 2014
VP33	JNTO01000000	CFSAN006132	36	Howard	MD	6/17/2013	stool	2,1	Haendiges et al., 2014
VP36	JNTP01000000	CFSAN006133	36	Baltimore County	MD	7/5/2013	stool	2,1	Haendiges et al., 2014
VP38	JNTQ00000000	CFSAN006134	36	Baltimore City	MD	7/16/2013	stool	2,1	Haendiges et al., 2014
VP40	JNTR00000000	CFSAN006135	36	Baltimore County	MD	7/21/2013	stool	2,1	Haendiges et al., 2014
VP42	JNTS00000000	CFSAN007460	36	Baltimore City	MD	8/7/2013	stool	2,1	Haendiges et al., 2014
VP43	JNTT00000000	CFSAN007461	36	Talbot	MD	7/31/2013	stool	2,1	Haendiges et al., 2014
VP30	JNTV00000000	CFSAN006130	36	Pringe George's	MD	6/2/2013	stool	2,1	Haendiges et al., 2014
VP46	JNTU00000000	CFSAN007462	36	Pringe George's	MD	8/27/2013	stool	2,1	Haendiges et al., 2014
VP5^*^	JNSY01000000	CFSAN007441	113	Calvert	MD	6/5/2012	wound	0,0	Haendiges et al., 2014
VP13^*^	JNTD01000000	CFSAN007446	678	Charles	MD	8/5/2012	wound	0,0	Haendiges et al., 2014
VP6^*^	JNTB01000000	CFSAN007444	677	Baltimore City	MD	6/25/2012	wound	0,0	Haendiges et al., 2014
VP15^*^	JNTF01000000	CFSAN007448	679	Out of State-PA	MD	8/3/2012	wound	0,0	Haendiges et al., 2014
VP3^*^	JNSV01000000	CFSAN007438	652	Anne Arundel	MD	6/8/2012	wound	0,0	Haendiges et al., 2014
VP11^*^	JNTA01000000	CFSAN007443	113	Somerset	MD	7/23/2012	ear	0,0	Haendiges et al., 2014
VP14^*^	JNTE01000000	CFSAN007447	162	Wicomico	MD	8/10/2012	ear	0,0	Haendiges et al., 2014

The isolates were organized by their isolation source.

a type of tdh or trh gene present in that strain by in silico reference mapping using CLC Genomics workbench. Example: 2,1 means tdh2 and trh1 types, respectively, and 0 means not present.

* Strains isolated from wound or ear.

**Table 2:** **Locally and globally diverse *V. parahaemolyticus* isolates genomes used for phylogenomic analyses available at the National Center for Biotechnology Information**.

**Isolate name**	**Accession no(s) WGS**	**CFSAN number**	**STs**	**State**	**Country**	**Isolation year**	**Source**	**References**
029-1(b)	JNTW00000000	CFSAN001611	36	OR	USA	1997	E	Haendiges et al., 2014
48057	JNTX00000000	CFSAN001612	36	WA	USA	1990	C	Haendiges et al., 2014
K1198	JNTY01000000	CFSAN001614	59	AK	USA	2004	E	Haendiges et al., 2014
10292	JNTZ00000000	CFSAN001617	50	WA	USA	1997	C	Haendiges et al., 2014
48291	JNUA00000000	CFSAN001618	36	WA	USA	1990	C	Haendiges et al., 2014
F11-3A	JNUB00000000	CFSAN001619	36	WA	USA	1988	E	Haendiges et al., 2014
NY-3483	JNUC00000000	CFSAN001620	36	NY	USA	1998	E	Haendiges et al., 2014
K1203	JNUD00000000	CFSAN001173	59	AK	USA	2004	E	Haendiges et al., 2014
98-513-F52	JNUE00000000	CFSAN001160	34	LA	USA	1998	E	Haendiges et al., 2014
10290	JNUF00000000	CFSAN001613	36	WA	USA	1997	C	Haendiges et al., 2014
10329	NZ_AFBW01000000	N/A	36	WA	USA	1998	C	Gonzalez-Escalona et al., 2011
RIMD 2210633	NC_004603, NC_004605	N/A	3	Osaka	Japan	1996	C	Makino et al., 2003
BB22OP	CP003973.1, CP003972.1	N/A	88	?	Bangladesh	1980	E	Jensen et al., 2013
AN-5034	ACFO00000000	N/A	3	?	Bangladesh	1998	?	Chen et al., 2011
AQ3810	AAWQ00000000	N/A	87	?	Singapore	1983	C	Unpublished
AQ4037	ACFN00000000	N/A	96	?	Maldives	1985	?	Chen et al., 2011
K5030	ACKB00000000	N/A	3	?	?	2005	?	Chen et al., 2011
PCV08-7	AOCL00000000	N/A	808	Selangor	Malaysia	2008	E	Tiruvayipati et al., 2013
Peru-466	ACFM00000000	N/A	3	?	Peru	1996	?	Chen et al., 2011
SNUVpS-1	AMRZ00000000	N/A	917	?	Korea	2009	E	Jun et al., 2013
V110	AQPJ00000000	N/A	809	?	China	2010	E	Liu and Chen, 2013
3259	AVOL01000000	N/A	479	?	USA	2007	C	Unpublished
949	AVPV01000000	N/A	3	?	USA	2006	C	Unpublished
NIHCB0603	AVOM00000000	N/A	3	?	Bangladesh	2006	C	Unpublished
NIHCB0757	AVPX01000000	N/A	65	?	Bangladesh	2006	C	Unpublished
VP-NY4	AVON01000000	N/A	3	?	India	1997	C	Unpublished
VP2007-095	NZ_AVOI01000000	N/A	631	FL	USA	2007	C	Unpublished
VP232	NZ_AVOJ01000000	N/A	3	?	India	1998	C	Unpublished
VP250	NZ_AVOK01000000	N/A	3	?	India	1998	C	Unpublished
VPCR-2010	NZ_AVPW01000000	N/A	308	?	USA	2010	E	Unpublished
12310	AYXP00000000	N/A	36	WA	USA	2006	C	Unpublished
3256	AZGS00000000	N/A	36	WA	USA	2007	C	Unpublished

N/A, not applicable; ?, unknown.

### DNA extraction and quantification

Genomic DNA from each isolate was isolated from overnight cultures using the DNeasy Blood and Tissue Kit (QIAGEN, Valencia, CA). The quality of the DNA was checked using a NanoDrop 1000 (Thermo Scientific, Rockford, IL) and the concentration was determined using a Qubit double-stranded DNA HS assay kit and a Qubit 2.0 fluorometer (Life Technologies, Grand Island, NY), according to each manufacturer's instructions.

### Whole genome sequencing, contig assembly, and annotation

The genomes of the historical isolates were sequenced using 200 bp reads chemistry and the 36 MD outbreak isolates were sequenced using 300 bp reads chemistry, using an Ion Torrent (Thermo Scientific) sequencer, according to manufacturer's instructions, at approximately 20X coverage (Haendiges et al., 2014). Genomic sequence contigs were *de novo* assembled using CLC Genomics Workbench (QIAGEN). These draft genomes were annotated using the NCBI Prokaryotic Genomes Automatic Annotation Pipeline (PGAAP, http://www.ncbi.nlm.nih.gov/genomes/static/Pipeline.html) (Klimke et al., 2009).

### *In silico* MLST phylogenetic analysis

The initial analysis and identification of the isolates was performed using an *in silico V. parahaemolyticus* MLST approach, based on information available at the *V. parahaemolyticus* MLST website (http://pubmlst.org/vparahaemolyticus). Seven loci (*dnaE, gyrB, recA, dtdS, pntA, pyrC*, and *tnaA*), described previously for *V. parahaemolyticus* (Gonzalez-Escalona et al., 2008), were used for MLST analysis. The same *V. parahaemolyticus* MLST database was also used to assign numbers for alleles and STs.

### Assignment to clonal complexes

The program goeBURST v1.2.1 was used to identify CCs among the isolates (http://goeburst.phyloviz.net) (Francisco et al., 2009). To qualify as members of the same CC, isolates must share at least 5 of the 7 alleles used in the *V. parahaemolyticus* MLST scheme (Feil et al., 2004). Those STs that differ by two alleles are considered double locus variants (DLV), and those which differ at only a single locus are single locus variants (SLV).

### Phylogenomic analysis and targeted SNP analysis

We performed two phylogenetic analyses using the software kSNP v2 (Gardner and Hall, 2013) (kmer length = 31). The first one included a broad SNP analysis of all the genomes included in the analysis, including local, national and international isolates, and was performed to provide an evolutionary context for all the *V. parahaemolyticus* isolates used for the analysis. The second was conducted to provide a more detailed phylogenetic relationship among the isolates found in each individual cluster and were targeted for more refined SNP analyses. Our goal was to recover variations unique to each respective lineage. To do this, we created a matrix that included only core SNPs (i.e., SNPs that were present in all genomes) for each lineage, using the reference-free SNP- finding program, kSNP v2 (Gardner and Hall, 2013) (kmer length = 31). Maximum-likelihood phylogenies for each core SNP matrix were then constructed within the kSNP, labeled with the number of unique SNPs present in every descendant of that node. Draft assemblies were used to detect SNPs using the program, kSNP. Filtering was applied to eliminate detectable horizontal gene transfer such as mobile elements and paralogs, but we were not able to filter for genome recombination. Although important, reconstructing the reticulate evolutionary history of *V. parahaemolyticus* was outside the scope of this research effort.

Using the kSNP approach offers several advantages. First, the kSNP approach to gathering reference-free SNPs for downstream phylogenetic analysis has been effective for microbial-scale phylogenomic analyses (Sahl et al., 2013; Timme et al., 2013), Second, these kSNP analyses are not as vulnerable to assembly errors as other methods because the putative SNPs are extracted from kmers (in our present analysis, one SNP per 25 mer), effectively eliminating any influence of assembly bias. Third, the algorithm behind SNP identification is especially conservative about common pyrosequencing errors, such as the homopolymeric runs commonly observed in data from 454 and Ion Torrent sequencing procedures: if an insertion or deletion is present in a region, the resulting 25-mer will no longer match the other homologous kmer regions, thus that SNP will be missing in that taxon. Additionally, kSNP omits kmers where all SNPs are in the center of a homopolymer repeat (Gardner and Slezak, 2010). These features make our approach conservative and prevent repeat regions from becoming a factor in our analyses: any k-mers comprising such regions will fail to be unique, and therefore will not be used.

### Nucleotide sequence accession numbers

The draft genome sequences for all 46 *V. parahaemolyticus* strains in our analyses are available in GenBank under the accession numbers listed in Tables 1, 2.

## Results and discussion

### Maryland *V. parahaemolyticus* outbreak strains and *in silico* MLST

Our MLST analyses provide a broad look at the diversity of *V. parahaemolyticus* clinical samples collected during 2010–2013 in the state of Maryland. These 34 *V. parahaemolyticus* strains isolated from patients—stool (80%), ear (6%), and wound (14%) were sequenced using Ion Torrent. A quarter of these strains (25%) came from an outbreak in the summer of 2013. All the reported cases occurred during the warmer months between June and August (Table 1).

*In silico* MLST (Gonzalez-Escalona et al., 2008) identified 13 different STs, showing that the sporadic outbreaks in Maryland were caused by highly diverse *V. parahaemolyticus* strains. Seven STs were novel (STs: 651, 652, 653, 677, 678, 679, and 896) and represented strains that have only been found (so far) in Maryland (Table 1). The most prevalent STs were ST36 (28%), ST631 (22%), ST3 (8%), ST113 (8%), and ST653 (6%). The rest of the STs were represented by a single strain.

**Figure 1:**
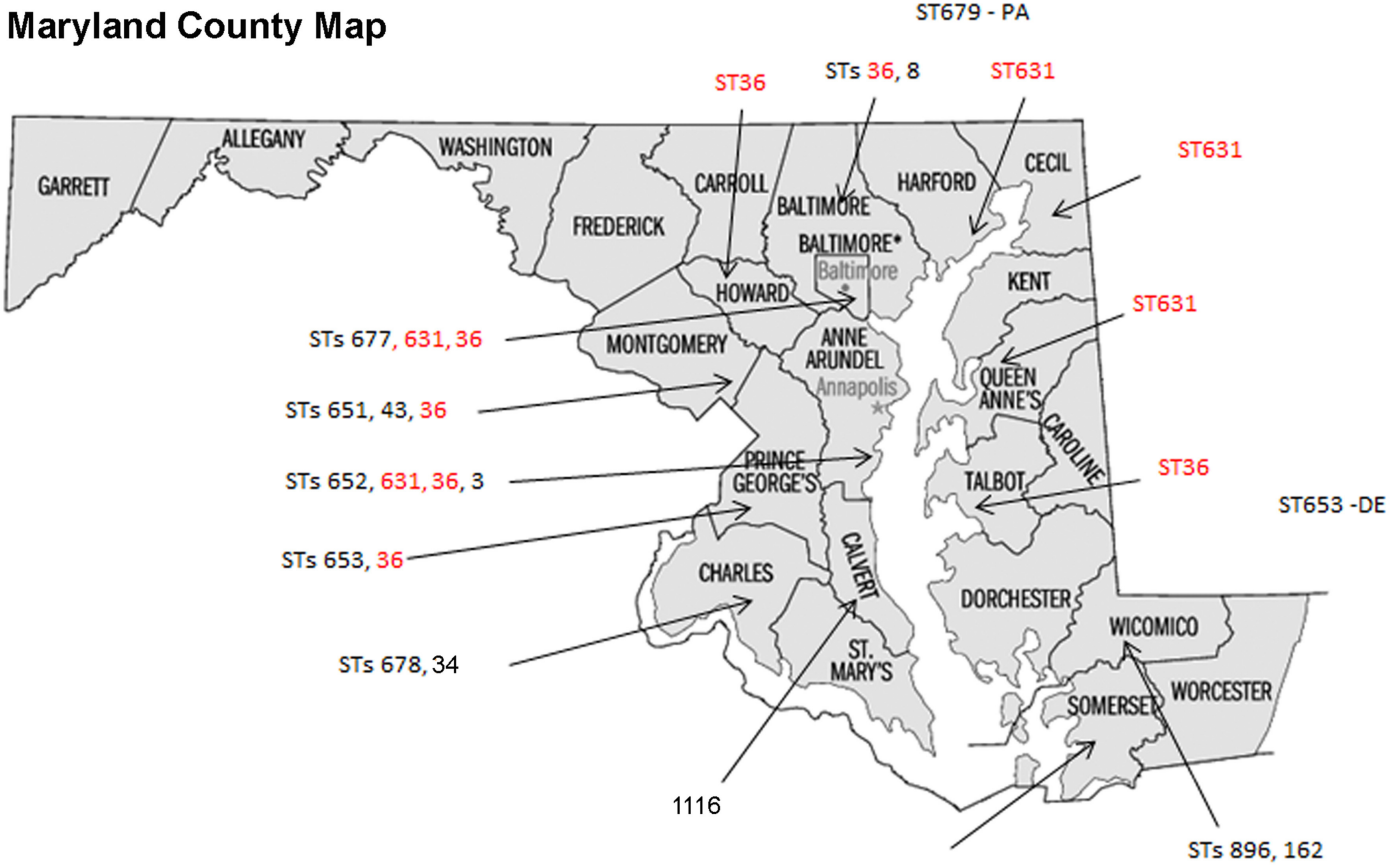
**Sequence types (STs) isolation locations on the state of Maryland, USA**. STs found in more than one location are in red fonts.

Some of the identified STs were isolated from more than one location, more than one person, and in different years (Table 1 and Figure 1); most of the illnesses associated with these isolates occurred in MD coastal counties (Figure 1). Interestingly, most of the cases on 2013 (88%) were caused by ST631 (31%) and ST36 (56%). This suggests these two STs are either more prevalent and/or have replaced the endemic *V. parahaemolyticus* populations in this region. Additional surveys of pathogenic *V. parahaemolyticus* in MD could confirm or clarify our theories.

**Figure 2:**
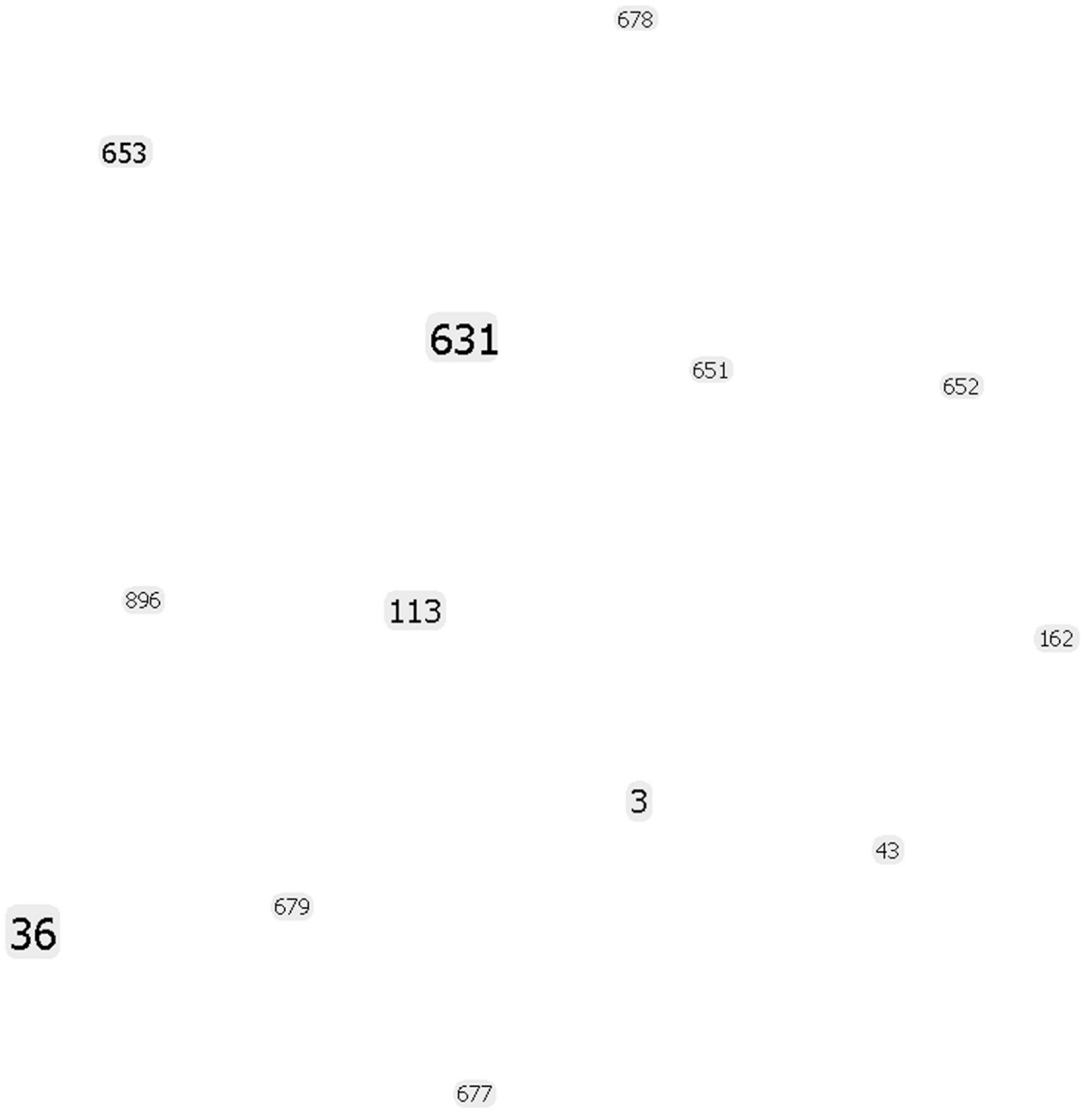
**goeBURST population snapshot using the *in silico* MLST analysis of the strains sequenced in this study indicating the absence of any clonal complex and the relative frequency of each ST**. Font sizes varied according to the frequency of the ST.

Our eBURST analysis (goeBURST) showed that the MD isolates do not form any clonal complexes and were not related to each other (Figure 2). Therefore, the outbreaks during this period in MD were caused by *V. parahaemolyticus* belonging to different populations. However, some findings suggested we had reached the limits of the MLST method: the ST36 observed in MD could not be distinguished from ST36 from the West Coast by this method. It is at this point we must turn to the higher discriminatory power of WGS analyses.

### Phylogenomic analysis

**Figure 3:**
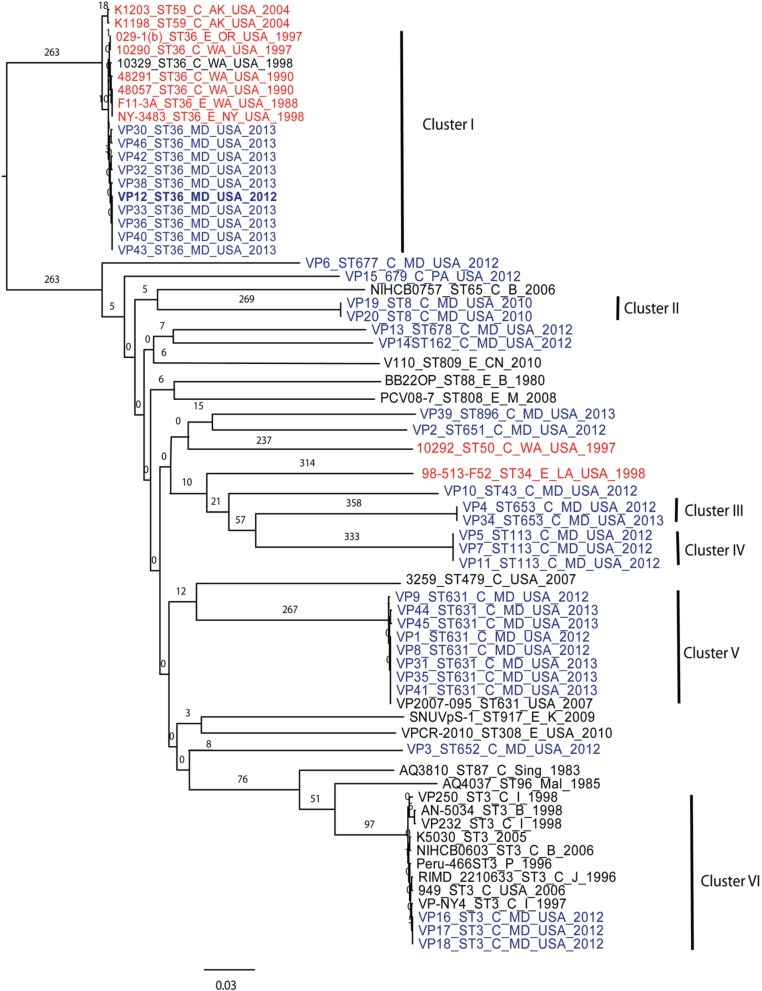
**Phylogenetic clustering analysis of the outbreak strains of *V. parahaemolyticus*, Maryland, 2012–2013, by whole-genome SNP analysis**. A k-SNP (Timme et al., 2013) analysis was performed. The maximum-likelihood tree shows *V. parahaemolyticus* MD outbreak isolates (in blue fonts), historical US strains sequenced in this study (in red fonts) and other related but unassociated *V. parahaemolyticus* strains (retrieved from NCBI, black fonts). Maximum-likelihood phylogeny was constructed from a 46,963 SNP matrix. Samples are annotated as follows: strain name, sequence type (ST), source (C, clinical; E, environmental), country of isolation (B, Bangladesh; CN, China; M, Malaysia; Mal, Maldives; USA, United States of America; Sing, Singapore; I, India, P, Peru; and K, Korea), and year when the samples were collected. All gene alignments of the SNPs observed in the entire dataset as well as in each individual cluster are available on request from the authors.

Using 7 *V. parahaemolyticus* genomes available from NCBI (www.ncbi.nlm.nih.gov/genome/?term=vibrio parahaemolyticus) and the 10 genomes from historical US *V. parahaemolyticus* strains (Table 2), we determined the phylogenetic relationships among the MD strains using a whole-genome SNP analysis (Figure 3). As can be seen from Figure 3, *V. parahaemolyticus* is a highly diverse microorganism, documented by the high number of SNPs defining each branch, and a low-resolution tree is obtained when using this entire genome database. Nevertheless, a similar clustering to the one achieved by MLST analysis can still be observed for the Maryland *V. parahaemolyticus* strains, where the same STs were also grouping at the genome level. Five clusters of related strains were identified, cluster I (ST36), cluster II (ST653), cluster III (ST113), cluster IV (ST631), and cluster V (ST3).

Besides Maryland ST36 strains (which appear in blue text on the Figure 3), Cluster I also contained strains that were isolated on the West Coast (1988–2004) (represented by the red text in Figure 3) and are part of CC36 (Gonzalez-Escalona et al., 2008). In this broad phylogeny, little differentiation can be observed among these ST36 strains. The same can be observed for most other clusters. But this broad phylogeny does allow us to confirm the diversity of populations causing the outbreaks in MD during that 2 year period.

**Figure 4:**
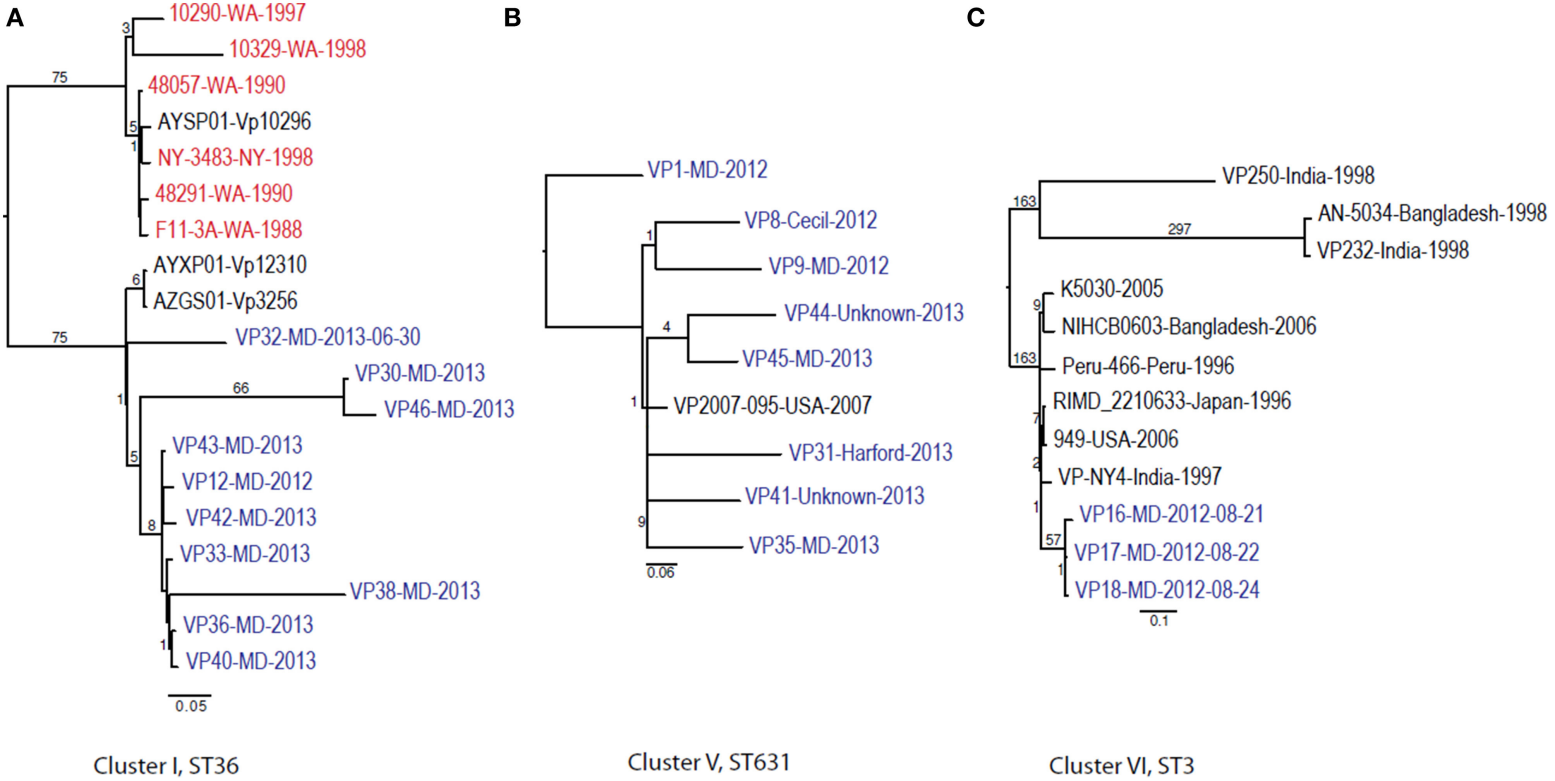
**Maximum-likelihood trees for each individual *V. parahaemolyticus* outbreak cluster identified in Figure 3 and other related (if any) but unassociated *V. parahaemolyticus* strains (retrieved from NCBI)**. Maximum-likelihood phylogeny was constructed from a 347, 931, and 88 core SNP matrix for clusters I, V, and VI, respectively. **(A)** Cluster I, **(B)** cluster V, and **(C)** cluster VI. Samples are annotated as in Figure 3. All gene alignments of the SNPs observed in the entire dataset as well as in each individual cluster are available on request from the authors.

Given these patterns, we then used a cluster-based analysis that enabled us to identify relationships among strains that would not have been found by examining the entire dataset (Figure 4). Each individual cluster (when more than three strains) was analyzed separately. This type of analysis helped us to clearly distinguish strains in ST36 from MD (2012–2013) from those originating on the West Coast (1988–1998). These groups differed by at least 150 SNPs (Figure 4A). Interestingly, MD strains were similar to ST36, also from the West Coast, of US but from a later time point: 2006–2007 (strains 12310 and 3256). This clearly indicates that ST36 strains have evolved in the last decade and the new ST36 strains from the West Coast are more similar to the ST36 from MD (2012–2013) than the historical ST36 from the West Coast. Also, it seems that the outbreak in 2013 in MD of ST36 was also composed by strains from at least three different subgroups within lineage ST36. One subgroup contained the strain responsible for the cases in 2012 and most 2013 cases (VP43, 12, 42, 33, 36, 40, and 38), and the other two subgroups were composed of one (VP32) and two strains (VP30 and VP46). These differences in subgroups within lineage ST36 could have arisen from adaptations to surviving in different oysters/habitats and/or to the introduction of new variants.

This is why WGS-SNP analyses provide clearer insights than earlier methods: many of these strains were unable to be distinguished from one another by MLST and PFGE analysis. The general understanding of *V. parahaemolyticus* evolution suggests that the typical mechanism is recombination, with a recombination ratio estimated at 2.5:1 and 8.8:1 by allele and site, respectively (Gonzalez-Escalona et al., 2008). Example of this are observed among strains belonging to CC345, where all known variants (SLV) of the ancestral ST type of the CC (ST345) have arisen by recombination, mainly in the *recA* allele (González-Escalona et al., in press).

Cluster II and Cluster III were composed by two and three strains each, respectively; no other genomes have been reported for those STs, therefore we could not use a targeted SNP analysis to further analyze those strains. Nevertheless, these clustered with other US strains (Figure 3) indicating that they are probably strains endemic to the US. Strains belonging to ST113 were isolated from wound, ear, and stool in MD, suggesting that Clusters II and III are composed of local strains. ST113 was first described for a *V. parahaemolyticus* strain isolated from oysters in Louisiana state in 2007 (Johnson et al., 2009).

The second most numerous ST observed among the MD outbreak strains was Cluster IV (ST631): these strains were highly similar, suggesting that they came from the same region 2 years in a row. In our analyses, the Cluster IV MD strains also clustered with a strain isolated in FL in 2007 (Figure 4B). If we fit this data together with the fact that ST631 had initially been reported through a strain isolated in Prince Edward Island (Canada) in 2009 (Banerjee et al., 2014), we have reason to believe that ST631 is a regional ST, endemic to the East Coast.

Finally, Cluster V (ST3), included the MD 3 strains recovered from an outbreak that caused six illnesses during the summer of 2012 (Haendiges et al., 2014). These strains had previously been characterized by Haendiges et al. (2014), using a different phylogenetic tool (BIGSdb genome comparator tool). Due to homopolymer errors (inherent to Ion Torrent sequencing), no further resolution could be observed among strains belonging to Cluster V, so we could not determine whether the strains causing the outbreak were the same or not. The other analyses performed in our study were not impacted by such errors, since the miscalls were pruned from the analysis and only true high quality SNPs were included. With this targeted SNP based analysis, these strains were undistinguishable and can be clearly separated from other ST3 strain which genome were available at NCBI by at least 57 unique SNPs and can conclusively be called as being undistinguishable and probably the same strain and coming from the same source (Figure 4C). Therefore, this targeted SNP analysis can be used for outbreak detection caused by a specific *V. parahaemolyticus* strain.

### Presence and absence of *tdh* and *trh* genes and their relation to etiology

There are five different subtypes of *tdh* genes that differ by sequence (*tdh1* through *tdh5*); these subtypes share 96–98% identity (Nishibuchi and Kaper, 1990). In the case of *trh* genes there are two recognized subtypes (*trh*1 and 2), which share 84% identity (Kishishita et al., 1992). Most of the clinical MD *V. parahaemolyticus* analyzed carried *tdh* and/or *trh* genes (72%) (Table 1). Ten isolates (28%) were negative for *tdh* and *trh*, three (8%) were only *tdh* positive, four (11%) were only *trh* positive, and the remaining 19 (53%) isolates contained both genes.

It was unusual to find that 28% of these clinical *V. parahaemolyticus* did not carry *tdh* or *trh*, since these strains are usually considered non-pathogenic (Nishibuchi and Kaper, 1995; Blackstone et al., 2003; DePaola et al., 2003). There have been some recent reports of *V. parahaemolyticus* without either of these two pathogenicity markers causing sporadic cases of illness (Jones et al., 2012; Banerjee et al., 2014) and having these 10 genomes may help researchers discover what makes these strains pathogenic. Jones et al. (2012) reported that 27% of their clinical isolates collected across US from July 2006 to November 2007 were *tdh/trh* negative while Banerjee et al. (2014) reported the same genotype for 4% of the clinical *V. parahaemolyticus* collected in Canada during 2000–2009. Furthermore, the majority of the *tdh/trh* negative strains (70%) were isolated from either wound or ear infections (Table 1). This further indicates that these two markers may not be necessary for human illness to occur (Broberg et al., 2011), although it is possible that these patients had some kind of immune deficiencies that could make them more prone to infections (chronic liver disease), as observed for *V. vulnificus* (Kim et al., 2014). The actual percentage of *tdh/trh* negative clinical strains is currently hard to estimate and might depend on several factors including sample size, *V. parahaemolyticus* endemic populations, type of infection, as well as errors caused by sporadic outbreaks that often go unnoticed or underreported (Scallan et al., 2011).

Most of the *tdh* and/or *trh* positive strains carried *tdh*2 and *trh*1 combination. Most clinical US strains carry both *tdh/trh* (Okuda et al., 1997a; Jones et al., 2012; Paranjpye et al., 2012; Turner et al., 2013) while pandemic strains carried *tdh*1 and *tdh*2 (Okuda et al., 1997b; Matsumoto et al., 2000; Makino et al., 2003; Gonzalez-Escalona et al., 2005). Among the MD *V. parahaemolyticus* strains, only three were both *tdh*1 and two positive and these were identified as belonging to the pandemic clonal complex and were ST3 (Haendiges et al., 2014). Only two MD strains carried exclusively *trh*2. A study of environmental *V. parahaemolyticus* in Norway also identified strains containing only the *trh* gene that were also *trh*2, however those strains belonged different STs than the one observed in this study (Ellingsen et al., 2013). The availability of these genomes would help in future analysis in determining which other genes besides *tdh* and *trh* are necessary for pathogenesis in this marine bacterium.

### Distribution of T3SS and other genomic regions in MD *V. parahaemolyticus* clinical strains

We performed an *in silico* analysis of each strain's genome using CLC Genome Workbench software (QIAGEN) for 24 known regions described for pandemic strains, including both T3SS, T3SS1 and 2α (Boyd et al., 2008) and well as for T3SS2β (Park et al., 2000), containing the *ure* genes and *trh* gene and described previously for strains carrying both *tdh* and *trh* genes (Supplementary Table 1). All strains carried NK, T3SS-1, Osmotolerance (chromosome I), Gametolysin, Osmotolerance (chromosome II), CPS, Type I secretion, Type I pilus, Multidrug efflux, and Ferric uptake. Most strains lacked all the pathogenicity islands described for the pandemic strain RIMD2210633 (VPaI1-7). Four other MD strains carried either VpaI1 (VP10) or VpaI2 (VP2, and VP14), although carrying a sequence different from that of the pandemic strain. Of the nine MD strains lacking *tdh* or *trh* gene, six carried a T6SS different from the pandemic, for the presence/absent of the other elements or PIs were very similar to the strains carrying *tdh*, *trh*, or *tdh/trh* strains. Seven of these strains were isolated from wound or ear infections. Since *tdh/trh* and T3SS2s were described as needed for producing infections at the intestinal level [because *tdh/trh* causes intestinal fluid secretion as well as cytotoxicity in a variety of cell types (Raimondi et al., 2000) while TTSS2 has a role in enterotoxicity (Park et al., 2004)], then these strains could have caused infections by a rather opportunistic way than by an actual infection as observed for gastroenteritis. These strains still possess TSS1 which is known for being involved in the cytotoxicity (Park et al., 2004). A more detailed study could reveal additional elements we did were not able to assess in this study; such analyses will certainly shed more light on the infection mechanisms of these unusual *V. parahaemolyticus* strains.

## Conclusions

Our study used WGS to provide a high resolution investigation of genetic diversity and relationships of clinical *V. parahaemolyticus* strains isolated in the state of Maryland during 2012–2013 period in comparison to those isolated from other geographical locations. Having an archive of *V. parahaemolyticus* genomes allowed us to perform a phylogenetic analysis of strains and revealed that strains causing outbreaks in MD were highly diverse, genetically distinct, and clearly different from other regional and global strains. Despite the geographic breadth of our strains, we are aware that our study captured only a fraction of the actual number of *V. parahaemolyticus* strains causing illness in the state of Maryland, due in part to the sporadic nature of outbreaks, the low number of strains recovered per outbreak, and the fact that many outbreaks are never investigated (Scallan et al., 2011).

We suggest using a similar approach during future outbreaks to analyze the identity of suspect *V. parahaemolyticus* strains and their relationship to strains causing previous outbreaks. Our WGS analysis of *V. parahaemolyticus* strains highlights the need for a genome database for *V. parahaemolyticus* to improve traceback and responses to outbreaks. We have created a new BioProject (PRJNA245882) at NCBI, spearheaded by FDA-CFSAN and the state of Maryland's DHMH, in which we are going to continually deposit new available genomes of *V. parahaemolyticus* causing outbreaks that would improve detection of new outbreak strains, track the emergence of new clonal strains in geographical regions where these strains are not endemic, and will be used in source tracking. We suggest and encourage the scientific community to deposit further *V. parahaemolyticus* genomes from their respective countries in centralize databases, such as NCBI, in order to improve source tracking.

### Conflict of interest statement

The authors declare that the research was conducted in the absence of any commercial or financial relationships that could be construed as a potential conflict of interest.
